# Skeletal muscle quality as an independent associated factor of oxygen utilization efficiency in interstitial lung disease: a cardiopulmonary exercise testing study

**DOI:** 10.1186/s12890-026-04529-z

**Published:** 2026-08-13

**Authors:** Kento Kaneko, Masahiro Ishizaka, Tsuyoshi Hara, Kohei Chiba, Kenya Takei, Fumiaki Ishikawa, Haruka Fujioka, Keita Sakamoto, Nobuyasu Awano, Minoru Inomata, Hitomi Takahashi, Takehiro Izumo

**Affiliations:** 1 Department of Rehabilitation Technology, Japanese Red Cross Medical Center, 4-1-22 Hiroo, Shibuya-ku, Tokyo, 150-8935 Japan; 2 Graduate School of Medicine and Health Sciences, International University of Health and Welfare, 4-1-26 Akasaka, Minato-ku, Tokyo, 107- 8402 Japan; 3 Department of Physical Therapy, School of Health Sciences, International University of Health and Welfare, 2600-1 Kitakanemaru, Otawara, 324-8501 Tochigi Japan; 4 Nutrition Department, Japanese Red Cross Medical Center Tokyo, 4-1-22 Hiroo, Shibuya-ku, Tokyo, 150-8935 Japan; 5 Department of Physical Therapy, School of Health Sciences, Fukushima Medical University, 10-6 Sakaemachi, Fukushima, 960-8516 Japan; 6 Department of Respiratory Medicine, Japanese Red Cross Medical Center, 4-1-22 Hiroo, Shibuya-ku, Tokyo, 150-8935 Japan

**Keywords:** Interstitial lung disease, Lower-limb PhA, Cardiopulmonary exercise testing, O_2_ pulse, Muscle quality

## Abstract

**Background:**

Reduced exercise capacity in patients with interstitial lung disease (ILD) is associated with poor prognosis. Oxygen pulse (O_2_ pulse) reflects exercise circulatory efficiency, but its determinants at submaximal exercise remain unclear. Phase angle (PhA) has recently emerged as a surrogate marker of muscle quality.

**Objective:**

To investigate the association between lower-limb PhA and submaximal O_2_ pulse at the ventilatory threshold (VT), which reflects daily physical activity independent of maximal volitional effort, in patients with ILD.

**Methods:**

This retrospective study analyzed 111 patients with ILD enrolled in an educational hospitalization program (April 2024–March 2026). Among these, 35 patients completed cardiopulmonary exercise testing (CPET). Lower-limb PhA was assessed via bioelectrical impedance analysis. The association between PhA and O_2_ pulse at VT was evaluated using Pearson correlation and multiple regression analysis adjusted for age, %FVC, and tricuspid regurgitation velocity (TRV).

**Results:**

The analyzed CPET sub-cohort (*n* = 35) had a mean age of 69.0 ± 8.4 years, 74.2% male, and %FVC of 85.3 ± 18.1%. Lower-limb PhA was significantly correlated with O_2_ pulse at VT (*r* = 0.629, *p* < 0.001). In multiple regression analysis, PhA remained independently associated with O_2_ pulse (β = 0.748, *p* = 0.001), with a model R^2^ of 0.433.

**Conclusions:**

Lower-limb PhA is an independent associated factor of O_2_ pulse in patients with mild-to-moderate ILD. Given the cross-sectional and retrospective nature of this study, these findings do not establish causality but suggest that muscle quality may be a relevant clinical indicator that warrants further prospective and interventional investigation.

## Introduction

In patients with interstitial lung disease (ILD), reduced exercise tolerance not only limits physical activity but also directly leads to a significant deterioration in quality of life (QOL) and an increased risk of death [1–2]. Traditionally, this decline has been attributed primarily to impaired gas exchange and restrictive ventilatory dysfunction associated with pulmonary fibrosis, as well as increased cardiac workload resulting from a reduction in pulmonary vascular bed volume [3–4].

Recent findings suggest that even in cases of mild ILD where hypoxemia is not observed at rest, compensatory tachycardia—an increase in heart rate to compensate for increased pulmonary vascular resistance—occurs during exercise stress testing [3, 5]. This condition not only places an excessive burden on the cardiopulmonary system but also leads to reduced oxygen transport efficiency due to insufficient stroke volume (SV). The O₂ pulse in cardiopulmonary exercise testing (CPET) is the product of stroke volume (SV) and the arteriovenous oxygen difference (a–vO₂ difference) [6]; while it is a critically important indicator for evaluating exercise efficiency in ILD patients, its determining factors have not been fully elucidated [5].

Traditionally, a reduced O₂ pulse in patients with ILD has been thought to primarily reflect limitations in stroke volume (SV) expansion due to pulmonary circulatory impairment (limitations of cardiac pump function). However, based on Fick’s equation, the O₂ pulse is an indicator that encompasses not only cardiac function but also changes in the arteriovenous oxygen difference (a–vO₂ difference) of peripheral skeletal muscle.

In recent years, attention has been focused on the decline in skeletal muscle function that occurs in ILD patients even before the onset of respiratory failure; however, the extent to which the quality of peripheral skeletal muscle contributes as a determinant of the O₂ pulse has not yet been fully elucidated.

In interstitial lung disease (ILD), where changes in pulmonary vascular structure progress irreversibly, peripheral skeletal muscle has garnered attention as a modifiable factor for maintaining exercise efficiency [7–8]. While the influence of factors such as skeletal muscle mass (Skeletal Muscle Mass Index: SMI) has been examined in the past, in recent years, the Phase Angle (PhA) obtained through Bioelectrical Impedance Analysis (BIA) has been regarded as an important indicator reflecting cell membrane integrity and muscle cell density—that is, muscle quality [9–10].

Previous studies have reported that Phase Angle (PhA) is an independent determinant of exercise tolerance, physical activity, and even prognosis in patients with interstitial lung disease (ILD) [1–11]. In this context, pulmonary rehabilitation targeting peripheral skeletal muscle has garnered attention as a crucial therapeutic strategy to improve exercise capacity, particularly because pulmonary structural changes progress irreversibly. However, contemporary pulmonary rehabilitation programs often apply generalized protocols, and the exact physiological mechanisms linking muscle quality to exertional circulatory efficiency remain poorly understood. Specifically, it remains unclear how Lower-limb Phase Angle (Lower-limb PhA) affects oxygen transport and utilization efficiency (O_2_ pulse) during exercise, independent of established clinical indicators such as pulmonary and cardiac function. Physiologically, since cycle ergometry primarily imposes metabolic and mechanical demands on the lower extremities, utilizing segment-specific lower-limb PhA—rather than a whole-body average—allows for a direct evaluation of localized cellular integrity where the exertional stress is actively applied. Assuming that muscle quality is associated with oxygen efficiency, therapeutic intervention targeting muscle quality from the early stages of the disease could potentially contribute to optimizing exercise capacity and reducing exertional cardiopulmonary stress. Therefore, the purpose of this study was to clarify the relationship between Lower-limb PhA and circulatory efficiency (oxygen pulse) during exercise in mild ILD, thereby providing a physiological basis for developing personalized rehabilitation strategies in this population.

## Materials and methods

### Study design and participants

This study was a retrospective observational study of patients with interstitial lung disease (ILD) who participated in an educational hospitalization program at our hospital. The study population consisted of patients aged 18 years or older who were diagnosed at our hospital’s Interstitial Pneumonitis Center between April 2024 and March 2026 and underwent a 5-day educational hospitalization.

Of the 118 potentially eligible patients, seven were excluded due to: (1) inability to undergo bioelectrical impedance analysis (BIA) (*n* = 4); (2) incomplete clinical data (*n* = 2); or (3) physician-directed exclusion from the 6-minute walk test (*n* = 5) (Note: numbers are not mutually exclusive). Consequently, 111 patients were included in the final analysis.

**Fig. 1:**
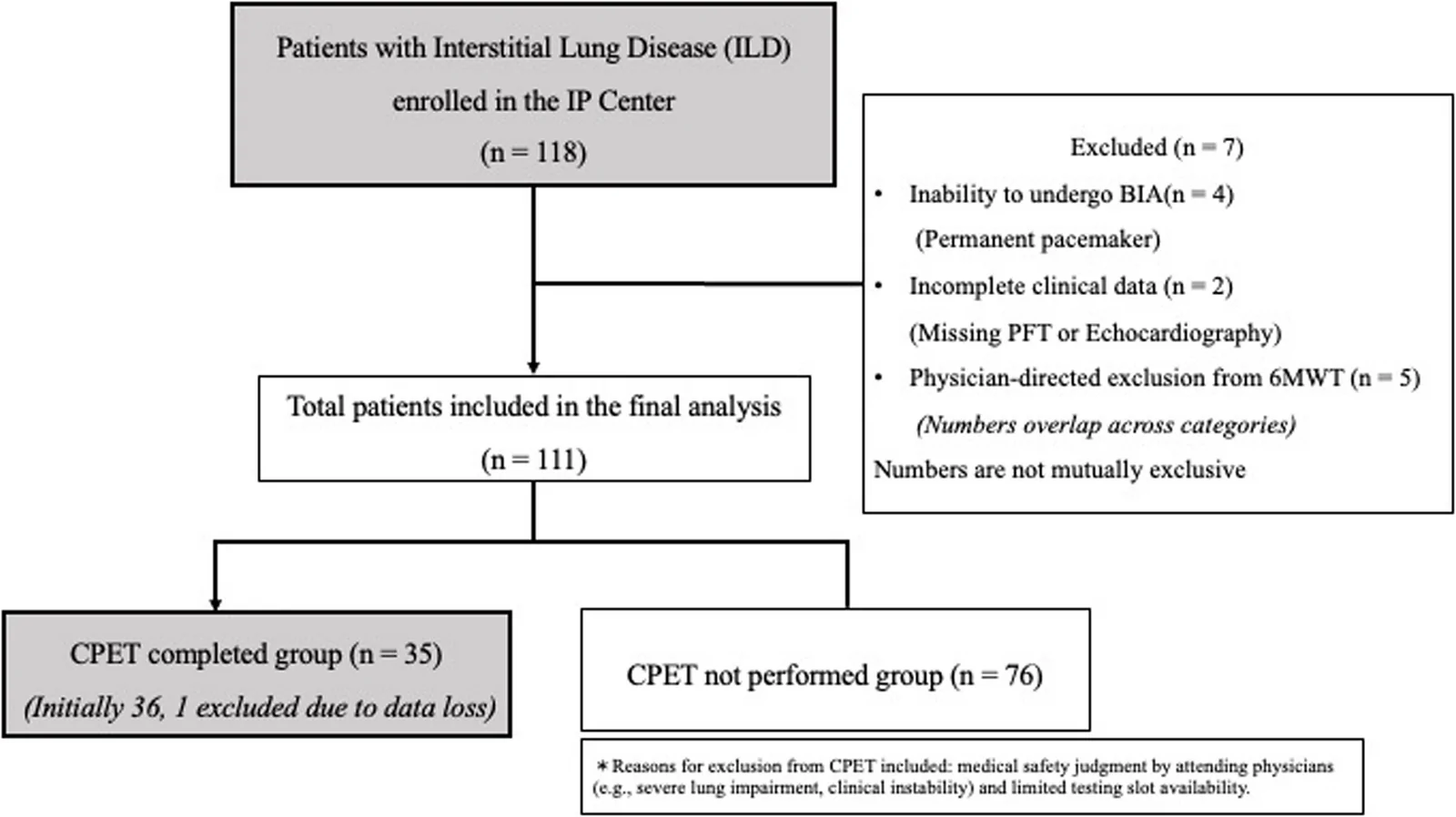
Patient selection process.The flow diagram illustrates the rigorous patient selection process. Of the 118 potentially eligible patients with interstitial lung disease (ILD), 7 patients were excluded due to: inability to undergo bioelectrical impedance analysis (*n* = 4), incomplete clinical data (*n* = 2), or physician-directed exclusion from the 6-minute walk test (*n* = 5). The remaining 111 patients were included in the final analysis. Among them, 35 patients who successfully completed both cardiopulmonary exercise testing (CPET) and bioelectrical impedance analysis (BIA), following the exclusion of 1 patient due to data loss, were allocated to the CPET group for detailed physiological assessment

Among these, 36 patients underwent cardiopulmonary exercise testing (CPET). One patient was excluded due to data loss during the test, and consequently, 35 patients who successfully completed both CPET and bioelectrical impedance analysis (BIA) were assigned to the CPET group for detailed physiological assessment. The selection for CPET was based on the attending physician’s clinical judgment regarding safety and clinical stability. In our institutional clinical practice, patients considered appropriate for CPET generally met the following practical criteria: (1) clinical stability with no acute exacerbation within the past 3 months, (2) mild-to-moderate restrictive impairment (typically %FVC ≥ 50%), (3) absence of severe resting hypoxemia requiring long-term oxygen therapy, and (4) the physical and cognitive ability to safely perform cycle ergometry. CPET was primarily utilized to investigate exertional dyspnea or exercise-induced desaturation in patients with relatively preserved resting lung function. This clinical selection process inherently introduced an indication bias toward a milder cohort. The detailed patient selection process is illustrated in Fig. 1.

In this study, “mild-to-moderate ILD with relatively preserved exercise capacity” was explicitly defined based on both clinical and physiological criteria. Clinically, patient severity was stratified using the ILD-GAP index (Stage I or II). Physiologically, this target population was characterized by preserved lung volumes and exercise capacity, with the CPET sub-cohort exhibiting a mean percent predicted forced vital capacity (%FVC) of 85.3 ± 18.1%, a percent predicted diffusing capacity for carbon monoxide (%DL_CO_) of 85.6 ± 31.8%, and a 6-minute walk distance (6MWD) of 497.5 ± 72.6 m.

All clinical and physiological assessments were systematically completed within a strict timeline of within 7 days of each other. Specifically, to comply with institutional reimbursement regulations, CPET was performed in the outpatient setting immediately prior to admission, while BIA (Day 3), the 6MWT (Day 2), echocardiography, and pulmonary function testing were completed immediately upon admission. Furthermore, because this educational hospitalization program strictly targets outpatients with clinically stable ILD, no patients experienced acute exacerbations, respiratory symptom changes, or medication adjustments during this brief multi-day testing window, ensuring that all measurements captured an identical, stable clinical state.

### Patient demographics

Patient demographics, including age, sex, height, weight, and BMI at admission, were recorded. The GAP index was used to assess severity. Blood chemistry tests included white blood cell count (WBC), hemoglobin (Hb), C-reactive protein (CRP), albumin (ALB), KL-6, SP-D, and B-type natriuretic peptide (BNP).

### Respiratory function assessment

The parameters measured in the respiratory function tests were forced vital capacity (FVC), forced expiratory volume in one second (FEV1.0%), diffusing capacity of the lung for carbon monoxide (DL_CO_), and vital capacity (VC). The assessment date was the most recent test before admission.

Echocardiographic Assessment.

During echocardiography, the following parameters were measured: left ventricular end-diastolic diameter (LVDd), left ventricular end-systolic diameter (LVDs), ejection fraction (EF), the ratio of early diastolic mitral inflow velocity to early diastolic mitral annular velocity (E/e’), and maximum tricuspid regurgitation velocity (TRV). The assessment was performed on the day of admission.

### Assessment of motor function and physical performance

On admission, walking speed over 4 m, grip strength, and knee extension muscle strength were measured. Grip strength was measured twice with each hand while seated on the edge of a chair using a GRIP-D T.K.K. 5401 (Takei Kiki Kogyo Co., Ltd., Tokyo, Japan), and the higher value was recorded. Knee extension muscle strength was measured using a handheld dynamometer (µTas F-1, Anima Co., Ltd., Tokyo, Japan) while seated on the edge of the bed, with two measurements taken for each leg, and the higher value was recorded. For knee extension muscle strength, both the absolute value and the weight-normalized value (kgf/kg) were calculated.

### 6-Minute walk test

The 6-Minute Walk Test (6MWT) was administered on the second day of hospitalization. The measured parameters included walking distance, minimum SpO₂, maximum heart rate, and the Borg scale. The test was conducted by a team of three physical therapists with expertise in pulmonary rehabilitation (PR).

### Body composition assessment

Body composition was measured on the third day of hospitalization using the BIA method. The MC-780 A-C (Tanita Corporation, Tokyo, Japan) was used for this measurement. To ensure standardized conditions, the assessment was performed in the afternoon under controlled hydration and fasting protocols; all patients were instructed to avoid eating, vigorous exercise, and excessive fluid intake immediately prior to the measurement, with all PhA values evaluated at a standardized frequency of 50 kHz. The measured parameters included muscle mass, protein mass, basal metabolic rate, fat-free mass, SMI, and Lower-limb PhA as an indicator of muscle quality. Specifically, “Lower-limb PhA” was defined as the segment-specific phase angle automatically calculated and derived directly by the BIA device software, rather than a manual calculation or an average of both legs.

### Cardio-pulmonary exercise testing

CPET was performed by a cardiologist and a clinical laboratory technologist. The equipment used included the MLX-1000 monitoring system and the Strength Ergo 8 V2 (Fukuda Denshi Co., Ltd., Tokyo, Japan). The test was conducted using an incremental ramp protocol on a cycle ergometer. Following a 3-minute rest period and a 3-minute warm-up at 0 W, the workload was increased at a constant rate of 10 W/min. Oxygen consumption (V̇O_2_) and carbon dioxide production (V̇CO_2_) were continuously measured via breath-by-breath respiratory gas analysis.

The ventilatory threshold (VT) was determined using the V-slope method, and the result was independently confirmed by the attending cardiologist. The O₂ pulse at VT was calculated by dividing oxygen consumption by heart rate. Testing was performed on admission. The decision to perform the CPET was based on the attending physician’s clinical judgment. Each test was conducted during a clinically stable period shortly before or after admission, and the influence of the timing of measurement is considered to be minimal.

### Statistical analysis

Statistical analysis was performed using IBM SPSS Statistics version 31.0 (IBM Corp., Armonk, NY, USA).

Continuous variables are presented as mean ± standard deviation or median [interquartile range]. To compare patient characteristics between the CPET group and the non-CPET group, Student’s t-test, Mann–Whitney U test, or chi-square test was used, depending on the data distribution.

The normality of all continuous variables was assessed using the Shapiro-Wilk test and visual inspection of Q-Q plots to confirm that the assumptions for parametric testing were met.

The association between Lower-limb PhA and exercise tolerance as well as clinical indicators was evaluated using Pearson’s correlation coefficient.

Furthermore, multiple regression analysis was performed to examine the independent association of Lower-limb PhA with O₂ pulse at VT. The independent variables—age, TRV, and %FVC—were selected a priori as essential clinical confounders based on their physiological relevance to oxygen pulse, specifically aging, pulmonary vascular load, and restrictive ventilatory impairment, respectively. Sex and BMI were not included in the multivariate model because of the limited sample size and the need to avoid model overfitting.

With the O₂ pulse at VT as the dependent variable, we performed an analysis using the forced entry method, with Lower-limb PhA, age, TRV, and %FVC as independent variables. Multicollinearity was assessed using the variance inflation factor (VIF), with a threshold of less than 2.5. For all statistical analyses, the significance level was set at less than 5%.

## Results

### Patient characteristics

Of the 118 patients initially screened, seven patients were excluded based on the following overlapping criteria: inability to undergo BIA due to a permanent pacemaker (*n* = 4), incomplete clinical data due to missing PFT or echocardiography (*n* = 2), and physician-directed exclusion from the 6MWT (*n* = 5). Importantly, all four patients who were unable to undergo BIA were also included in the physician-directed exclusion from the 6MWT cohort, resulting in a unique total of seven excluded individuals.

This study ultimately included 111 patients with ILD; of these, 35 who underwent CPET were included in the sub-group analysis. The patient characteristics for the overall group, as well as for the CPET group and the non-CPET group, are shown in Table 1.

**Table 1:** Baseline characteristics and physiological parameters of patients with ILD

Variable	Total (*n* = 111)	CPET group (*n* = 35)	Non-CPET group (*n* = 76)	*p*-value
Demographics				
Age (years)	71.6 ± 9.6	69.0 ± 8.4	72.8 ± 9.9	0.052
Sex, male, n (%)	73 (65.7%)	26 (74.2%)	47 (61.8%)	0.261
Height (cm)	161.3 ± 9.4	162.5 ± 12.9	160.8 ± 9.1	0.421
Weight (kg)	59.0 ± 14.4	61.3 ± 12.9	57.9 ± 15.3	0.228
BMI (kg/m²)	22.5 ± 4.1	23.0 ± 3.3	22.1 ± 4.5	0.243
Diagnosis				
IPF, n (%)	20 (18.0%)	4 (11.4%)	16 (21.0%)	-
fHP, n (%)	27 (24.3%)	11 (31.4%)	16 (21.0%)	-
IIPs (excluding IPF), n (%)	12 (10.8%)	7 (20.0%)	5 (6.5%)	-
CTD-ILD, n (%)	15 (13.5%)	7 (20.0%)	8 (10.5%)	-
PPFE, n (%)	16 (14.4%)	2 (5.7%)	14 (18.4%)	-
Others, n (%)	21 (18.9%)	4 (11.4%)	17 (22.3%)	-
Severity				
^†^GAP index	2 [1–4]	1 [1–4]	2 [1–4]	0.001
Exercise				
6MWD (m)	420.4 ± 117.6	497.5 ± 72.6	381.7 ± 119.4	0.001
Lowest SpO_2_ during 6MWT (%)	89.9 ± 5.7	90.7 ± 5.5	89.2 ± 5.9	0.214
Peak heart rate during 6MWT (bpm)	114.3 ± 16.8	114.2 ± 18.4	114.8 ± 16.3	0.876
Symptoms				
^†^Borg scale at rest	0 [0–5]	0 [0–4]	0 [0–5]	0.010
^†^Borg scale at end of exercise	4 [0.5–10]	3 [0.5–10]	4 [0–9]	0.057
Physical				
4-meter gait speed (m/s)	1.03 ± 0.2	1.16 ± 0.2	0.97 ± 0.2	0.001
Handgrip strength (kg)	25.8 ± 7.8	27.6 ± 7.1	25.0 ± 8.1	0.093
Isometric knee extension force (kgf)	29.1 ± 12.3	33.5 ± 14.2	27.0 ± 10.9	0.020
Isometric knee extension force / BW (kgf/kg)	0.50 ± 0.1	0.53 ± 0.17	0.46 ± 0.14	0.031
Blood Test				
CRP (mg/dL)	0.40 ± 0.5	0.11 ± 0.10	0.50 ± 0.66	0.001
WBC (/µL)	7111.8 ± 2454.6	6315.5 ± 1725.6	7572.5 ± 2681.2	0.004
Hb (g/dL)	13.4 ± 1.4	13.7 ± 1.2	13.2 ± 1.5	0.055
Alb (g/dL)	4.0 ± 0.4	4.0 ± 0.4	3.9 ± 0.4	0.092
KL-6 (U/mL)	1025.0 ± 823.0	924.1 ± 128.4	1068.4 ± 913.8	0.318
SP-D (ng/mL)	242.3 ± 183.1	207.2 ± 198.9	248.5 ± 175.0	0.168
BNP (pg/mL)	37.8 ± 37.9	31.0 ± 41.1	40.5 ± 36.5	0.248
Pulmonary function				
%FVC (%)	80.5 ± 21.4	85.3 ± 18.1	77.7 ± 23.0	0.067
%FEV1 (%)	82.9 ± 19.8	85.7 ± 17.3	81.3 ± 21.3	0.261
%DL_CO_ (%)	74.9 ± 27.2	85.6 ± 31.8	67.7 ± 22.0	0.005
VC (%)	77.7 ± 20.9	83.1 ± 17.0	74.7 ± 22.7	0.038
Cardiac function				
LVDd (mm)	44.3 ± 5.4	46.2 ± 5.1	43.4 ± 5.4	0.010
LVDs (mm)	27.8 ± 4.2	29.3 ± 3.9	27.1 ± 4.2	0.012
EF (%)	67.7 ± 6.1	66.8 ± 5.3	68.0 ± 6.5	0.295
E/e’	9.30 ± 3.4	9.1 ± 2.8	9.4 ± 3.7	0.640
TRV (m/s)	2.40 ± 0.4	2.2 ± 0.3	2.5 ± 0.4	0.001
BIA				
Lean body mass (kg)	43.0 ± 9.1	44.9 ± 9.4	42.0 ± 9.1	0.146
Muscle mass (kg)	40.8 ± 8.7	42.5 ± 8.9	39.8 ± 8.6	0.148
Protein mass (kg)	11.2 ± 3.1	11.1 ± 3.0	11.2 ± 3.3	0.810
Basal metabolic rate (kcal)	1180.1 ± 248.3	1235.8 ± 251.0	1153.8 ± 249.5	0.123
SMI (kg/m²)	6.9 ± 1.1	7.2 ± 1.1	6.7 ± 1.1	0.032
Lower-limb PhA (degree)	4.5 ± 0.9	4.8 ± 0.8	4.3 ± 0.9	0.003

Data are presented as mean ± SD or †median [interquartile range]. *p*-values were calculated using the Student’s t-test or ^†^Mann-Whitney U test

*Alb* albumin, *BIA* bioelectrical impedance analysis, *BMI* body mass index, *BNP* B-type natriuretic peptide, *CRP* C-reactive protein, *DL*_*CO*_ diffusing capacity of the lung for carbon monoxide, *FEV*_*1*_ forced expiratory volume in 1 s, *FVC* forced vital capacity, *Hb* hemoglobin, *HHD* hand-held dynamometer, *GAP index* interstitial lung disease-gender, age, and physiology, *KL-6* Krebs von den Lungen-6, *LVDd* left ventricular end-diastolic diameter, *LVDs* left ventricular end-systolic diameter, *PhA* phase angle, *SMI* skeletal muscle mass index, *SP-D* surfactant protein-D, *SpO*_*2*_ peripheral oxygen saturation, *TRV* tricuspid regurgitation velocity, *WBC* white blood cell count, *6MWD* 6-minute walk distance

Compared to the non-CPET group, the CPET group had significantly higher 6MWT scores, knee extension strength, and %DL_CO_, and significantly lower TRV. Additionally, the CPET group showed significantly higher Lower-limb PhA. No significant differences were observed for the other parameters.

The comprehensive functional and physiological profiles obtained from the CPET for the CPET group (*n* = 35) are summarized in Table 2. Notably, the ventilatory threshold (VT) was successfully identified in all 35 participants (100%). The mean peak oxygen uptake (V̇O_2_) was 23.3 ± 6.4 mL/min/kg, and the mean V̇E/V̇CO₂ slope was 27.3 ± 6.8, reflecting the baseline exercise capacity and gas exchange efficiency of this sub-cohort. (Table 2)

**Table 2:** Cardiopulmonary exercise testing parameters in the CPET group (*n* = 35)

Exercise Capacity & Work Rate	
Peak V̇O_2_ (ml/min/kg)	23.3 ± 6.4
Peak V̇O_2_ (% predicted)	101.0 ± 24.9
Peak work rate (W)	86.8 ± 32.7
Work rate at ventilatory threshold (W)	43.7 ± 18.1
Ventilatory threshold	
V̇O_2_ at ventilatory threshold (ml/min/kg)	14.2 ± 2.9
V̇O_2_ at ventilatory threshold (% predicted)	97.9 ± 20.2
Cardiovascular & Chronotropic Responses	
Heart rate at ventilatory threshold (bpm)	100.1 ± 16.1
Peak O_2_ pulse (ml/beat)	11.0 ± 3.8
O_2_ pulse at ventilatory threshold (ml/beat)	8.8 ± 2.9
Ventilatory & Gas Exchange Efficiency	
V̇E/V̇CO₂ slope	27.3 ± 6.8
OUES	2031.0 ± 615.2
ΔV̇O₂/ΔWR (ml/min/W)	11.7 ± 2.8

Data are presented as mean ± standard deviation

*Abbreviations* *V̇O₂* oxygen uptake, *VT *ventilatory threshold, *WR* work rate, *HR* heart rate, *bpm* beats per minute, *V̇E/V̇CO₂ slope* minute ventilation to carbon dioxide output slope, *OUES* oxygen uptake efficiency slope, *ΔV̇O₂/ΔWR* slope of oxygen uptake increase per unit workload increase

### Relationship between lower-limb PhA and O₂ pulse

**Fig. 2:**
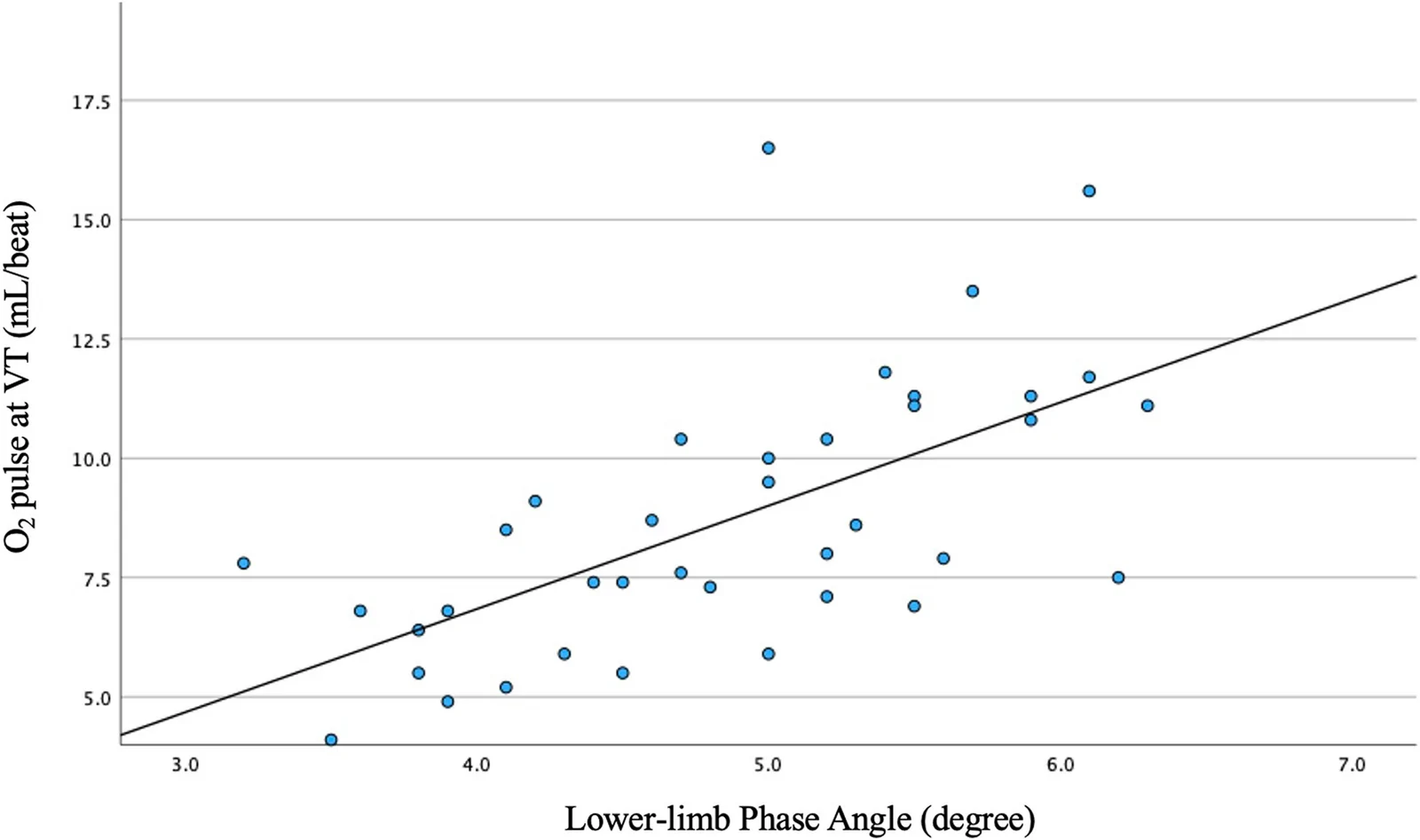
Correlation between Lower-limb PhA and O₂ Pulse at Ventilatory threshold A significant positive correlation was observed between Lower-limb PhA and O₂ pulse at VT (*r* = 0.629, *p* < 0.001, *n* = 35). The regression line indicates a consistent linear relationship between skeletal muscle quality and exercise efficiency

In 35 subjects who underwent CPET, the Lower-limb PhA showed a significant positive correlation with the O₂ pulse at the ventilatory threshold (VT) (*r* = 0.629, *p* < 0.001) (Fig. 2). This relationship was visually confirmed as a consistent linear relationship.

### Multiple regression analysis

In a multiple regression analysis with the O₂ pulse at VT as the dependent variable, Lower-limb PhA was identified as an independent associated factor (β = 0.748, *p* = 0.001) (Table 3).

**Table 3:** Multiple regression analysis for variables associated with Oxygen Pulse at VT

Variable	β	B	SE	t	*p*-value	VIF
Lower-limb PhA (degree)	0.748	2.444	0.700	3.491	0.001	2.349
%FVC (%)	0.247	0.038	0.024	1.589	0.123	1.241
TRV (m/s)	0.213	1.898	1.741	1.090	0.285	1.947
Age (years)	0.117	0.039	0.061	0.629	0.534	1.774

Multiple regression analysis was performed using the enter method. The dependent variable was oxygen pulse at the ventilatory threshold (VT). The model significantly predicted oxygen pulse at VT (*R*^*2*^ = 0.433, adjusted *R*^*2*^ = 0.355, *p* < 0.001)

*Abbreviations* *%FVC* percent predicted forced vital capacity, *VT* ventilatory threshold, *B* unstandardized coefficient, *β* standardized coefficient, *SE* standard error, *TRV* tricuspid regurgitant velocity, *VIF* variance inflation factor

In contrast, age (β = 0.117, *p* = 0.534), %FVC (β = 0.247, *p* = 0.123), and TRV (β = 0.213, *p* = 0.285) showed no significant association (Table 3).

The overall model was significant (R² = 0.433, adjusted R² = 0.355, *p* < 0.001), and no multicollinearity was observed (all VIF < 2.5).

Furthermore, Lower-limb PhA exhibited the highest standardized regression coefficient among all variables included and was the primary associated factor of the O₂ pulse at VT.

## Discussion

The key finding of this study is that, in patients with ILD, Lower-limb PhA was significantly associated with the O₂ pulse during VT and emerged as an independent associated factor even after adjusting for age, lung function (%FVC), and cardiac function indicators (TRV). In particular, Lower-limb PhA exhibited the highest standardized regression coefficient among all explanatory variables, suggesting that it may be a major determinant of oxygen utilization efficiency during exercise.

### Physiological interpretation

The O_2_ pulse is generally interpreted as the product of stroke volume (SV) and the arteriovenous oxygen difference based on the Fick equation [6]. :

$$\:\mathrm{O}_2\:\mathrm{p}\mathrm{u}\mathrm{l}\mathrm{s}\mathrm{e}\:=\:\mathrm{S}\mathrm{V}\:\times\:\:(\mathrm{a}-\mathrm{v}\mathrm{O}_2\:\mathrm{d}\mathrm{i}\mathrm{f}\mathrm{f}\mathrm{e}\mathrm{r}\mathrm{e}\mathrm{n}\mathrm{c}\mathrm{e})$$

It reflects the efficiency of oxygen transport and utilization during exercise. The strong correlation between Lower-limb PhA and the O₂ pulse observed in this study suggests that lower-limb PhA, as a surrogate marker of muscle quality, may influence peripheral oxygen extraction capacity. PhA is an indicator reflecting cell membrane integrity and intracellular-extracellular water balance, and it has been suggested that it is closely associated with the functional state of muscle cells, namely mitochondrial function and capillary density [9–10]. The fact that Lower-limb PhA was an independent associated factor of the O₂ pulse in this study is compatible with the hypothesis that in cases maintaining good muscle quality, the limited SV during exercise might be supported by a high peripheral oxygen uptake rate (increased a–vO₂ difference).

Crucially, the robust association between Lower-limb PhA and O_2_ pulse, independent of resting TRV, indicates that skeletal muscle quality is a factor closely associated with circulatory efficiency, although the potential influence of dynamic pulmonary vascular responses and cardiac reserves during exercise cannot be entirely ruled out. This highlights the potential clinical value of physical therapists in managing the ‘circulatory-muscular axis’ to optimize exercise tolerance, particularly in the early stages of ILD.

### Comparison with existing research and new clinical perspectives

Regarding the clinical utility of exercise tests, the 6-minute walk distance (6MWD) is an established prognostic marker in advanced ILD [1]. However, its sensitivity in identifying early-stage physiological impairment can be limited, as patients with preserved lung function or milder disease frequently exhibit high walking distances that may mask subtle circulatory or ventilatory limitations [11]. Previous studies have also noted that 6MWD may not accurately reflect peak oxygen uptake on an individual level due to a large standard error of estimate [12].

In this study, even after adjusting for age, lung function (%FVC), and cardiac function indicators (TRV), Lower-limb PhA was identified as an independent associated factor of the O₂ pulse. Notably, this association remained significant even in our cohort where 6MWD was relatively preserved, suggesting that CPET-derived O₂ pulse may capture subtle circulatory inefficiencies that might not be fully apparent through 6MWD alone. This result takes the previously reported association between muscle quality and exercise tolerance [13] a step further, suggesting that the qualitative characteristics of skeletal muscle may be directly involved in oxygen utilization efficiency.

Furthermore, while previous studies have often focused on muscle mass (e.g., SMI), a key novelty of this study is its focus on PhA as a surrogate marker of muscle quality. Recently, it has been reported that surrogate markers of muscle quality, such as PhA and muscle ultrasonography, are superior to muscle mass in predicting clinical outcomes and mortality in patients with ILD [14]. It is a widely observed clinical phenomenon that differences in exercise tolerance can occur even when muscle mass is comparable; the results of this study support the possibility that differences in muscle quality are involved in this phenomenon [10].

In summary, the significance of this study lies in presenting a perspective that views PhA not merely as a nutritional or muscle mass indicator, but as a functional indicator associated with oxygen utilization efficiency during exercise.

The results of this study suggest that, in addition to pulmonary and cardiac function, peripheral skeletal muscle plays a role in the reduced exercise tolerance observed in ILD. While limitations in cardiac output due to pulmonary circulatory dysfunction are strongly implicated in the exercise limitations of ILD patients, it has been noted that peripheral skeletal muscle dysfunction is also a significant contributing factor [7]. These findings suggest that, during the progression of the disease—characterized by increased pulmonary vascular resistance and increased right ventricular workload—the oxygen utilization efficiency of skeletal muscle (as indicated by PhA) may play a crucial role in maintaining and compensating for the body’s overall exercise response.

In the early stages of ILD, the reduction in the pulmonary vascular bed leads to increased pulmonary vascular resistance. To compensate for limited stroke volume (SV) during exercise, marked tachycardia occurs; however, this chronic hemodynamic stress can accelerate right ventricular remodeling and disease progression. Crucially, based on our finding that Lower-limb PhA independently associated with O_2_ pulse, we propose the “physiological buffer hypothesis.” This concept suggests that optimizing peripheral oxygen extraction (a-vO_2_ difference) may act as a conceptual physiological buffer that interacts with compensatory tachycardia and potentially influence exertional cardiac workload. Although this physiological mechanism remains hypothetical and requires future longitudinal or invasive hemodynamic validation, muscle quality assessment via PhA and rehabilitation intervention initiated from the early stages of the disease represent a novel therapeutic strategy. Although longitudinal studies are required, optimizing systemic oxygen kinetics might serve as a potential therapeutic target to minimize exertional cardiopulmonary stress, even in patients who do not yet exhibit overt resting physiological impairment.

In particular, it has been reported that even when resting parameters show no obvious abnormalities, reduced oxygen utilization efficiency and changes in compensatory circulatory responses occur during exercise in the stages preceding the onset of pulmonary hypertension (PH) [15]. The fact that PhA was associated with the O₂ pulse independently of resting pulmonary and cardiac function indices in this study suggests that it may reflect these early physiological changes that cannot be fully captured by resting tests from the qualitative aspects of skeletal muscle (cell membrane integrity and metabolic activity).

Based on the above, qualitative assessment of skeletal muscle (PhA) is useful as an indicator that complements conventional pulmonary function tests and resting cardiac function evaluations to identify potential abnormalities during exercise. This provides a new perspective for elucidating the mechanisms underlying reduced exercise tolerance in ILD patients and for optimization strategies in pulmonary rehabilitation (PR) aimed at minimizing exertional cardiopulmonary stress.

### Clinical significance

The clinical significance of this study lies in its focus on muscle quality as a variable factor, in addition to pulmonary and cardiac function—which have traditionally been emphasized as associated factors of exercise tolerance. PhA can be assessed noninvasively and easily, and may be useful as an indicator for assessing a patient’s exercise potential in clinical practice. Furthermore, this suggests that exercise therapy and nutritional interventions aimed at improving skeletal muscle quality may improve oxygen utilization efficiency. This implies that there is room for intervention even in patients for whom improving cardiopulmonary function is difficult.

### Limitations

This study has several limitations. First, this is a single-center, retrospective observational study and does not establish a causal relationship. Second, the study population was limited to patients who underwent CPET, which introduces a substantial selection and indication bias. Because the selection was based on retrospective clinical decisions, Patients in the CPET group tended to have relatively preserved exercise capacity and milder disease compared to those who did not undergo testing. Therefore, our findings cannot be generalized to severe ILD cases or those with advanced respiratory failure, and apply strictly to patients with mild-to-moderate ILD who are capable of completing an incremental exercise test.

Third, due to the limited sample size (*n* = 35), the number of explanatory variables included in the multivariate model had to be restricted to avoid overfitting. Consequently, our model operates close to the conventional sample size threshold for multivariate analysis, which may potentially lead to overfitting and unstable coefficient estimates. Therefore, although sex and BMI, and specific ILD subtypes may potentially influence muscle quality and exercise physiology, they were not included in the final model. In addition, phase angle may be influenced by systemic inflammation, hydration status, nutritional condition, and medication use such as corticosteroids. Although body composition assessment was standardized, substantial residual confounding by these unmeasured or omitted variables cannot be completely excluded. Thus, this regression model supports an adjusted statistical association between lower-limb PhA and O₂ pulse, but should be interpreted as exploratory rather than definitive evidence of an independent physiological mechanism.

Fourth, because resting TRV does not fully reflect dynamic cardiac function during exercise, such as stroke volume reserve, right ventricular contractile reserve, or cardiac output kinetics, a central cardiovascular contribution to the O₂ pulse remains possible in this study. Direct measurements of stroke volume, cardiac output, or the arteriovenous O₂ difference during exercise would be necessary to definitively confirm the proposed peripheral mechanisms.

Nevertheless, from a practical standpoint, performing such invasive hemodynamics or specialized tissue-oxygenation monitoring is often challenging in routine, day-to-day clinical practice. Establishing lower-limb PhA as a non-invasive, easily accessible bedside indicator to screen for potential impairments in exercise pathophysiology holds substantial clinical utility for risk stratification and rehabilitation planning.

Additionally, due to the small sample size of the CPET cohort, we were unable to perform subgroup analyses to determine whether the association between lower-limb PhA and O₂ pulse varies across different ILD subtypes.

Fifth, although patients with ILD frequently present with sarcopenia, this study focused primarily on lower-limb PhA as a specific surrogate for muscle quality and did not comprehensively evaluate the overlapping impact of formal clinical criteria for sarcopenia (e.g., combining muscle mass, handgrip strength, and physical performance). Future prospective studies are warranted to clarify how lower-limb PhA interacts with the formal clinical diagnosis, staging, and longitudinal progression of sarcopenia in this population.

## Conclusion

This study demonstrated that Lower-limb PhA is a significant, independent associated factor of oxygen utilization efficiency during exercise in patients with relatively preserved exercise capacity, specifically those with mild-to-moderate ILD. Importantly, this association remains robust even after adjusting for pulmonary and cardiac function, suggesting that skeletal muscle quality is closely associated with oxygen utilization efficiency during exercise. Furthermore, assessing and maintaining lower-limb muscle quality holds clinical utility for identifying impaired exercise physiology. These findings highlight the importance of the peripheral muscle contribution in the management and rehabilitation of patients with mild-to-moderate ILD. and provide a valuable hypothesis-generating foundation that warrants confirmation in future longitudinal or prospective interventional studies.

## Data Availability

The datasets generated and/or analyzed during the current study are available from the corresponding author upon reasonable request.

## Abbreviations

ILD: Interstitial lung disease
CPET: Cardiopulmonary exercise testing
PhA: Phase angle
O2 pulse: Oxygen pulse
FVC: orced vital capacity
DL_CO_: Diffusing capacity of the lung for carbon monoxide
TRV: Tricuspid regurgitation velocity
6MWD: 6-minute walk distance
VT: Ventilatory threshold
BIA: Bioelectrical impedance analysis
SMI: Skeletal muscle mass index
